# The Oldest Dental Book

**Published:** 1891-08

**Authors:** 


### THE DENTAL REGISTER.

VOL. XLV.] AUGUST, 1891. [No. 8.

### TRANSLATION.

### The Oldest Dental Book.

Treatises upon the teeth and their diseases have been embodied in medical works of the most remote period; for ever since teeth were placed in the jaws of man they have been a source of pain and disease, the relief of which demanded the consideration of all who pretended to be curers of afflictions that make men sufferers.

So far as is known the first to consider the teeth of sufficient importance as to prepare a separate work upon them, was Peter Jordan, of Mayence, Germany, in the year 1532. The owner of the only copy of this little work, known to exist is Dr. H. J. McKellops, of St. Louis, whose extensive library contains many rare and choice works.

The following translation of this book, while it may not be adopted as a guide for modern practice, will prove interesting in showing the methods of our dental brethren of the sixteenth century. The title page is as follows: W. T.

DENTISTRY.

A treatise upon all kinds of infirmities and diseases of the teeth with, Many *wholesome* and *approved* medicines, taken from the books of Galen, Avicenne, Mesne, Cornelius Celsus and Pliny, with brief and useful *instructions* how to *preserve* them in good health and to extract the bad ones or their roots, *easily, safely* and without *pain*. The contents of this *Record* will furnish a remedy for every defect. Printed at Mayence, by Peter Jordan, in August, 1532.

CONTENTS OF THIS LITTLE BOOK.

First Chapter: When and how many Teeth Grow in Man.
Second Chapter: By what Causes the Teeth Are Ruined.
Third Chapter: How To Assist Children to Easy Teething.
Fourth Chapter: Of Sore Teeth.
Fifth Chapter: Of Hollow and Perforated Teeth.
Sixth Chapter; Of Corrosion of Teeth.
Seventh Chapter: Of Yellow and Black Teeth.
Eighth Chapter: Of Depressed Teeth.
Ninth Chapter: Of Wavering Teeth.
Tenth Chapter: Of Worms in the Teeth.
Eleventh Chapter: Of Ulcers and Stinking from Ulcerated gums.
Twelfth Chapter: Of Breaking out of the Bad Teeth.
Last Chapter: Of Preserving Good Teeth.

PREFACE.

The Lord Almighty, who is cognizant of every thing from its very beginning, in his inexpressible wisdom, has not vainly ordained that the teeth in brutes, as well as in mankind, should be the first preparers of food so that nature might be assisted in the preservation of the race. For if the stomach, from which the other parts of the body are nourished, become sick or weak, also the remainder of its members become so, even those parts which prepare our food, as a cook. Therefore it becomes us to take care of them and see that they do not suffer injury and thereby cause great impairment of the body. But the teeth are not only preparers of food, but are also given to man for speech, and so for government, especially the anterior ones, which at once in lovely sound, receive and echo the impulse of the tongue, and as Pliny says, "Create order and sweet civility." [Plinius libre 7. Cap. 16]. In falling out or being otherwise hurt and lost, they entirely prevent the pronunciation of some letters and words, to prevent which I have lately gathered the advice of old and experienced physicians in order that I might inform and advise our youth. I beg of my readers to accept the following lines good humoredly.

### THE FIRST CHAPTER.

### When and How Many Teeth Grow in Man?

In the first place it should be remarked that nature in producing teeth is quite erratic. Some (astonishing as it may seem) are born with teeth, as for instance, Marcus Curius, a renowned Roman, who was on this account called "Dentatus," which means a man with teeth. Also Cenus Pahirius, and some others. Some, instead of teeth have a full bone from which the teeth begin to sprout in the seventh month after birth, and from this on continue to grow until the age of thirty-two years. But in some they come with greater ease and at an earlier time than others. [Plinius lib. 11, Cap. 37].

Some teeth first appear in the twentieth year of age, with many, as Pliny says, (Plinius lib. 11, Cap. 37) "in the eightieth." They also fall out in old age, and with some regrow, as for instance, it is related of a Samothratian citizen in whose jaws teeth were growing again after having arrived at the age of one hundred and four years; as in the case of Timarchus Nikokleson, who had two well-developed jaws with teeth in them which never dropped out, but were completely worn away by use; this was also the case with his brother. There are also men who have teeth in their larynx, but this forms an exception and is to be counted among the marvels of nature.

### THE SECOND CHAPTER.

### THE CAUSES BY WHICH TEETH ARE RUINED.

Now, as the teeth are given to man for the purpose of chewing his food, as well as an instrument of speech, it becomes necessary for us to keep them, as far as possible, in good order and avoid all conditions that might invade and do them damage, and if any accident befall them (which often occurs) it becomes us to take care and apply a remedy before it gains too much headway, for if taken in the beginning its correction is easily accomplished, and medicines ought not to be applied too slowly. As a precaution every body, therefore, should after every meal have a care that the teeth be cleansed of adhering remnants of food, and the mouth be rinsed and purified with fine, pure water.

With all diligence foulness of the victuals taken in must be avoided, for it causes bad mist to arise from the stomach and seriously injures the teeth.

Also great care should be taken to prevent frequent vomitings which is followed with great damage to the teeth, for they may become broken, or ulcerous and unfit to even bite a straw. But where involuntary vomiting is the result (to assist nature and relieve the stomach) the mouth ought to be refreshed as soon as possible with rose-water or vinegar or some other purifying matter (rup. coloris dentium) repeatedly. (Mesne, Summa, Cap. 8, on the corruption of the teeth and color).

Eatables averse and, injurious to the teeth are to be avoided, as for instance dry figs, boiled honey, sweet garlic, (Avicenna ut Supra) (same advice as above) fat meat being tough, sour apples, vinegar from wood pears, milk and radish, all of which cause the teeth to become ulcerous.

One must also be careful not to bite on any hard stuff, such as hard nuts and the like, or any thing that in breaking injures the teeth.

All slimy, sticky and fat eatables are to be renounced, for they remain upon the teeth and not without damage. Also hot food is to be abstained from, for they do burn the teeth and can never be taken without doing damage to them.

Also very cold eatables and drinks must not be used for they cause very much pain, and besides it is admitted that they produce teeth caries as well as that of bone.

Moreover avoid taking quick-silver, or salves mixed therewith, and quite especially if putting them on a coal fire be careful not to inhale the fumes thereof so that they reach the teeth, and never take quick-silver as recommended by frauds, rubbing therewith red mint, and then placing the fingers in the mouth.

Also one ought not to go to sleep after loading the stomach, for this can only be done to the detriment of the teeth.

### THE THIRD CHAPTER.

### HOW TO ASSIST CHILDREN IN AN EASY GROWTH OF TEETH.

It has just been said that with human beings, as a rule, the teeth begin to crop out in the seventh month of their age and in the general course of nature they usually appear in connection with some accidental diseases accompanied with great pains, for which one may employ the following named useful means:

In the first place children whose teeth are often affected by soreness ought to be repeatedly bathed, and their gums after such bathing, should be gently rubbed with one finger anointed with warm chicken or goose grease. When the teeth are supposed to come out (shed) in order that they may not hurt too much (Avicenne, tert, fen, pri. lib. doct. 1 Cap. 2. Mesne proprie de gnatiœ dentium) take a hare's or rabbit's brain stuff, mixed with said fat and apply it outwardly. (Avicenne, lib. 2 de simpli capit 397).

Then take poplars, clover and camomiles boiled in water and while warm wash the head, neck and cheeks, (Mesne prima patri, Sectionis prima summa, 8 Capit. de gnatione dentin, Avicenne ut supra. (Mesne 1st Part, 1st Section 8th Chap. on ignition of teeth, Avicenne ut supra).

But after the teeth begin to make their appearance and come to sight, take fine suitable sheep's wool, from the lower part of the neck, and oil of camomile nicely warmed up, in which the wool is to be steeped; when so prepared apply it to the neck and jaw bones; this is very efficacious for children, making the growth of the teeth very easy. (Mesne ut supra).

It often comes to happen that with children above seven years of age when their teeth begin to fall out others crop out near them, wherefore the old one destined to be lost, and next to which the new one is sprouting, ought to be well cleansed and frequently shaken till it can be extracted. Then press the new one every day to the place where its predecessor has been standing and direct it until it comes to the exact place to the one extracted and in an equal line with the others. Then, if properly taken care of, it will remain in that position, otherwise the first tooth will turn back and the young one will be prevented from taking a straight position, nor can it by any means be brought back to its proper place.

### THE FOURTH CHAPTER.

### ON THE TEETH'S SORE DAYS.

What the sore days or pains of the teeth are nobody knows so well as he who has experienced them, and I hold that no greater pains than these can be found. They, once in a while, result from a bad condition and derangement of the veins entering into the teeth. Some are of the opinion that toothache is one of the hereditary diseases, innate to one by one's father or mother, which is probably not far from the truth, because now-a-days we see everywhere children mostly subject thereto, who have had parents both of whom have been suffering from it.

But relief from this pain, as told us by Johannes De Figo, is principally accomplished in three ways.

In the first place by well-governed eating and drinking by the one thereby affected. He must avoid every thing mouldering, foul and stinking, viz.: old dough, milk, cheese, fish roughly salted, dry or smoked meat and the like eatables as afore referred to.

Moreover, by removing the matter from which pain arises, and here the first change is to be affected by bleeding or cupping as Mesne directed, by cupping the artery or veins in the lips, doubly beneath the tongue, the liver vein, on the shoulders, below the neck, on the chin, nicely closing the gums with a fine pair of scissors letting the remaining blood ooze out; also the matter ought to be purged with the roseate electuary of Mesne or rhubarb pills, but if the pains are caused by cold, then by pills de fumio ferre, (Sine quibus effenolo) all to be found in a drug store, and which ought to be taken in accordance with the advice of an experienced body physician, as occasion and time may require.

Thirdly, pain may be removed by a manifold application of medicines and such as benumb and relieve it, which may be accomplished by applying either a simple remedy or one composed of several stuffs. The latter ones are again of two kinds, some which allay pain originating from heat, and some that arises from cold matter. These should be observed by every one.

If the pain should have its origin from cold take into the mouth according as may be agreeable, one, two or three doses of the following:

Take Bertram root, boil it in wine and keep it warm in the mouth, or take Bertram ginger and boil it in vinegar and use it in the same manner; or take some pepper and euforbium, crush them with honey and put them on the teeth: or take a part of the kernel of a peach, half a part of pepper, mix it up and apply to the teeth; or take Bertram ginger, pepper, louse root, or wolf weed, in equal portions, pound them together and rub the gums with it; or take seeds of onions, barley and the like, all in equal parts, compound them, making in cakes half as large as hazel nuts, then put one of them on a coal fire and cover it so that the smoke is bound to come up to the teeth which are causing pain; or take the seeds of mushrooms with a little Swiss ginger, Sandara, or Bertram, and pound well, with a little bag of a finger's length filled with it, apply to the sore tooth which will remove the pain; or take Bertram steeped for half a day in vinegar and apply to the sore tooth this will remove the pain.

On the other hand, if the pain is caused by heat use the folfowing, viz.: Take the root of the European sunflower with its white blossoms, a handful of roses, barley, sumach, in equal parts, tormentile and some other seeds, two quents (half ounces) and let them be stamped together, two handfuls of lettuce, one handful of lotus and boil together in two measures of rain water and one third part of vinegar, allow it to simmer down to one half and drain through a piece of cloth, and apply to the mouth on the side where the teeth are aching.

Avicenna says (Avicenna, lib. de simplicibus capit 66) "If you take serpent's skin and saturate with vinegar and place it to the mouth it will be good." Some add to the vinegar wine of pomegranates.

Or mushroom seed roots with oil of roses chewed with vinegar, also retained in the mouth, is good.

There may also be applied to the sick tooth metridatum nicolai, or take iron weed with its root, boil in wine to one-half its quantity and take it whilst warm into the mouth. But when the pains are heavy, and the teeth too much protrude take pepper, myrrh, burned beans, crush them to a powder, mix with it the white of an egg, make into a plaster by spreading upon a piece of cloth, place it upon the thin part of the cheek at the side where the pain exists. Mesne says, "Some medicines should be externally applied to the cheek where the pain is greatest, such as poplar, camomile flowers, clover, Grecian hay, linseed, seeds of parsnips, or lily roots and the like." These must be nicely crushed and prepared into a plaster, or they may be partly boiled in vinegar or wine and applied to the cheek. Outwardly oil of camomile may be used for rubbing in as well as several other oils.

Or take the seeds of mushrooms and of alum, four half-ounces, vertrum, three half-ounces, crush them to the utmost firm small cakes with vinegar the size of beans, and when suffering from toothache take one and rub it on the tooth and gums at the seat of pain, or put one in a little wine or vinegar, rubbing it in till dissolved and put it on the cheek and into the mouth where the suffering exists.

Or take pinks, calmus, cinnamon bark in equal parts, powder them as minutely as possible, add some brandy and let the mixture settle, then with it moisten a small piece of cloth and apply to the teeth.

Or oppoponacum dissolved in vinegar and boiled and held in the mouth is good for pains and cavities in the teeth.

Or take asafœtida, storacis mint, round hole-wort, opium, pepper, mushroom seed, ginger, in equal parts, crush them minutely together and mix with honey, and in case of need apply to the teeth which are aching.

Or hysop boiled together with honey and vinegar and inserted into the mouth relieves the pain.

Or frogs boiled in water and vinegar and holding the broth in one's mouth effects much. (Avicenna, lib. 2 de Simplicibus Cap. 596).

But if the cheeks begin to swell, this is an indication of vanishing pain, for the pain's basis then is retreating from the veins emptying into the teeth and the membrane or skin issuing from the brains and surrounding the teeth retires to the uppermost fleshy part, the cheeks and contiguous parts and inflates them by swellings for which the following is very good: Take half a handful of violet leaves, add to them dried grape berries and twenty gooseberries, a handful of barley a little crushed, moreover, an herb called oxen-tongue and liquorice, one ounce of each. Boil the whole together in rain water until it becomes reduced to half its original quantity. Then press and drain through a piece of cloth, then add two ounces of violet syrup (syrupi violarum). Repeatedly bathe the mouth with this and it will probably be an advantage.

Or rubbed and pounded parsley fastened to the hand relieves pain in the teeth, according to Mesne (ubi supra). Or parsley boiled in wine and a quarter of an ounce of olibano, and one third of an ounce of myrrh boiled to the density of honey, quickly relieves pain when applied to the suffering tooth.

Or corn-flowers boiled in vinegar and taken into the mouth assuage the pain as also does the root of mushroom seed; or the root of small thistles cooked in wine and kept in the mouth relieves the pain in the teeth.

Or frogs boiled in water or vinegar the broth to be taken into the mouth. (Avicenna, 2 lib. on simplicibus, Cap. 596).

Also garlic is very wholesome for ailing teeth especially if mixed with butter and externally applied.

Or rhubarb or worm-wood boiled in vinegar, wine or beer kept in the mouth relieves the pain, also the root of the large camomile, mustard, mulberry tree, as well as the bark of the maple, five-finger-weed, leaves of sunflowers, and the like, serve the purpose very well if taken singly, or two or three combined; or oil dregs also remove the pain.

### THE FIFTH CHAPTER.

### ABOUT PERFORATED OR HOLLOW TEETH.

Erosion is a disease gnawing the teeth whereby they become perforated or hollow, which most frequently attacks the buccals, especially if they are not well cleansed of adherent remnants of food, for if these are allowed to remain they become putrid, taking on a bad acid humidity which infects and corrodes the teeth which gradually increases until it entirely destroys them. They become rotten and painful and drop out one piece after another.

This suffering, as Mesne says, may be cured principally in three different ways (as Mesne says in the preceding chapter). First, by purging as referred to above; second, by neutralizing the matter which corrodes and hollows them out. This may be done by boiling flowers growing amongst wheat or rye in vinegar and taking them in the mouth; also ginger boiled in vinegar.

Thirdly, by removing the cavity, which may be done in two ways. Firstly, by removing the whole corrosion with a sharp chisel or knife or other fit instrument; scrape and clean and besides fill the small hole in the tooth with small lamellas of gold in order to preserve the adjoining one. Furthermore, apply proper medicine, such as gall-apples and wild-weed with which the tooth, after having been cleansed, may be filled.

Or take mushroom seed mixed with gummi storacis which must be ignited and inhaled through a funnel into the hollow tooth.

Or galbanum put in the perforated tooth alleviates the pain.

Or if the hollow tooth is filled with opoponnaco the pain will be removed.

Or put pounded torellas in the hollow teeth whereupon they shall drop out.

Some say that the teeth will never become hollow if one before breakfast every morning puts salt beneath his tongue until it melts away by itself.

For hollowness and pain of the teeth take pepper, bertram, galbanum and storacis in equal parts, pound together and apply to the teeth.

If hollow and corroded teeth are aching take the heart of a gall-apple and put it in the hole and it will relieve the pain.

For corrosion of teeth and their achings take pepper, bertram, nux vomica, especially the latter's milk (in Latin lacti-timalli) galbanum and storax, mix them together and therewith rub the teeth.

### THE SIXTH CHAPTER.

There does also attach to the teeth an ailment called congelation; properly meaning in Latin, "And it befalls the teeth." (As Johannes de Figo says). Sometimes from outward causes, sometimes from inward ones. From outward, when something sharp or sour is taken into the mouth, as for instance, sour-crout, sour wood apples, pears and other sour fruit. Inwardly, when bitter and sour humidities arise in the stomach and their eructations rise up into the mouth or gums, in such cases it is good to eat walnuts, hazel-nuts, or almonds, or mix together and pound them and rub the teeth with them, or you may take only one of the ingredients.

Or take, what in Latin is called, portulacca seeds and chew them in your mouth.

Or take some warm bread, roasted cheese, the yolk of an egg, which must be warm, add a little salt, mix together, and while warm, put it on the teeth.

Some under such circumstances used to eat laurels, which is not bad either.

### THE SEVENTH CHAPTER.

### FOR BLACK AND YELLOW TEETH.

Limosity is where white, yellowish or blackish slime (deposit) settles at the lower part of the teeth, by some called "tartar of cream."

Black and yellow teeth are caused by eating and consuming honey and other sweet matters, and he who wishes to retain white teeth should abstain from such things, and above all from sponges, mushrooms and all vegetables of that kind, or from wheat or other fruit.

The regular and natural shape of the teeth is preserved and the diseased ones restored by medicine which is drying and purifying, and possesses the power to rub off impurities and cleanse, as for instance, spumaris, salgemure, etc.

Ex. A powder to whiten teeth. Take the root of the wild gallow-weed close to the root where it is white, pound it, mix it into pellets with honey and dry in a stove which is not too hot.

Then take these pellets about half an ounce, rhubarb and cresses an eighth of an ounce, of each of carmecite and ligni-aloes a half ounce; prepare it into a powder and have the teeth rubbed therewith.

Or oil burnt with salt rubbed on the teeth will relieve and free them from their impurities.

Black teeth ought often to be scraped, smeared over and rubbed over with the following medicine: Take roses and one fourth part of as many gall-apples, or some other bitter herb, pound and compound, and rub the teeth with the mass.

Or take good burnt sulphur and brimstone two ounces of each, pound together and rub the teeth.

Or take raw dismembraned egg-shells and an equal part of brick, pound minutely together, and when it is to be used put in a spoon and pour some wine or vinegar over it and rub the teeth therewith and afterwards cleanse the mouth with some wine or pure water.

Or burn the stems of rosemarine to coal, pound and bind up in silk or other small pieces of cloth and frequently rub the teeth therewith. This will make the teeth white and remove the worms.

Another good powder for black teeth, and cause the wavering ones to grow fast, and cause a good smell from the mouth, is this: Take a quarter of an ounce of burnt alum, tartar of wine, red corals one half ounce each, coal rosemarine, cypress stems, sardel and sarcocolla, half an ounce of each, well pounded together and rub the teeth therewith.

### THE EIGHTH CHAPTER.

Dormitation is an ailment of the teeth acting in a like manner as it happens in a hand or foot when it is benumbed.

For this malady, if it arises from having taken into the mouth very cold substances such as snow, ice or cold water, then use the fat of a ram, applying it to the teeth.

Take a pound of good wine, rosemarine, salvey, flowers of camomile, half a handful of each, and strain; take it in the mouth warm and rinse it.

Or take pink, ginger, muscate and pepper, a little of each and put them in a little bag of a finger's length, then for half an hour in burnt wine, and then on the benumbed teeth.

### THE NINTH CHAPTER.

### FOR UNSTEADY TEETH.

This condition is called dentincommocro and relates to the teeth when they become loose and threaten to fall out prematurely. (Avicenna and Mesne, capitibus proprius). This may happen as a consequence from debility and weakness of the gums, or change of the bases sustaining and supporting the teeth, or it may happen when a vitiated fluid is pouring down from the head to the gums or roots of the teeth doing them damage; or else from diseases of the stomach when bad fluids arise therefrom and injure the gums. Mesne says (prima parte, sectionis prima, capiti propria) there are principally three ways for purging off humidity arising from the head, or the vapors from the stomach. First with medicine, each one, according to the greater or less degree of humidity. This is done by medicinal pills which clean the head.

Then dry off the humidity from the gums and apply convenient and appropriate medicine such as incense, bertram, ground onions and mint or louse weed, and the like, boiled in vinegar and held in the mouth by all of which the humidity will be dried up.

Thirdly, by applying medicine which will remove the humors from the gums and cleanse them. This is principally effected by mastix, if boiled with roses and grated flowers, then washing and rinsing the mouth therewith.

But when teeth become loosened or rickety from a beating, a fall or the like, they must be fastened to the firm and uninjured ones by a silk or gold thread in connection as Cornelius Celsus says, " with medicines that cause filling up and contraction, such as wine in which gall-apples have been drenched, or take half an ounce of gall-apples, myrrh one ounce, bark of pomegranate, common iris, in Latin called " yreos " half an ounce of each, boil together, rinsing the mouth and rubbing the gums therewith.

Or laudanum mixed with mastix to be used on the gums.

Or ashes of burnt hartshorn, or shaved hartshorn, rubbed on the teeth and the mouth washed with it, will fasten the teeth.

Also olives boiled in water will fasten the teeth, and no less will rinsing the mouth with oil of olives, fasten and improve hollow teeth.

Or take gall-nuts and the shells wherein acorns are growing, alumen seeds, peel of pomegranates, all in equal parts, pound them together and make a powder, put such powder in the mouth mornings and evenings and apply to the gums close to the lips, which is very good for fastening the teeth.

Or take roses, green plums, unripe berries of grapes, dry them in the sun, compound them into a powder, mix with honey and apply them to the rickety teeth.

Or take laudanum and powdered mastix, mix both with licio, apply to the teeth, and it will fasten them.

### THE TENTH CHAPTER.

### FOR WORMS IN TEETH.

For worms in teeth take seeds of the common henbane, leek and onion and boil together in vinegar, then take these seeds and pound them together with the fat surrounding the kidney of a goat; of this mass prepare small cakes of the size of beans, put under a funnel with a live coal and admit the smoke to the affected teeth. This will kill the worms therein.

Or take a little verdigris and twice as much honey, mix well and rub it on the teeth.

### THE ELEVENTH CHAPTER.

### FOR ULCERS, STENCH AND ROTTENNESS OF TEETH

If the gums are affected by ulcers thoroughly press them out with the fingers. If this should prove of no avail use a delicate and sharp cutting instrument to slit them open and allow the matter to ooze out, and then wash the mouth frequently with vinegar or wine boiled up with honey.

If the gums have a bad smell take some cinnamon, cloves, white frankincense and rub the teeth therewith. But if they become rotten take a quarter of an ounce of alum, half an ounce of honey, mix together and with it rub the gums. Or ground onions together with vinegar and licio juice kept in the mouth invigorates the teeth and removes the rottenness (sponginess) of the gums.

But where rottenness of the gums is gaining headway (Mesne ubi supra) it is advisable in the first place to purge the body, then thoroughly cleanse and dry out all putridity of the affected part with vinegar and squills until sound flesh begins to grow. If the rottenness penetrates the root and cleansing causes much pain it may be assuaged in the first place by rose oil, the white of an egg or milk, whereupon burn off all puridity with copper iron or red hot gold, and then apply to the burnt spot some butter with rose oil, soon after which incarnate with Egyptian liniment to consolidate, etc.

### THE TWELFTH CHAPTER.

### HOW TO EXTRACT UNSOUND TEETH.

If the pain can not by any means be assuaged, then in order that the other teeth may not become affected extraction must be resorted to, which must be performed by an expert in this line (Mesne in the proper chapter on Extraction of the Teeth) for great injury may be done by extracting a tooth in an improper manner. Therefore it ought not to be done at the greatest point of pain but at the time when it begins to diminish. Then the master with a suitable instrument should free the sick tooth from the gums so as not, at the same time tear them off, and aside from the pain inflict other incidental troubles. The gums being now profoundly separated from the tooth it must be well shaken so as to become quite rickety. Then extract in a gentle manner, and not in a hurry, so as to avoid breaking, splintering or distorting the jaw bone, as occurs once in a while by unexperienced men, especially when the tooth is standing in the upper jaw, for it is very liable to injure the eyes if any tooth be extracted in an unwise manner.

But if a tooth be hollow or perforated it ought to be filled with lead, tin, silver, or whatever is fit for the purpose, so that when it is taken by the tongs it may not break or collapse.

It must be drawn straight and forward and not to be bent aside, otherwise the root might break and the jaw bone be hurt.

After the tooth has been in this way extracted diligently examine whether or not some small pieces of bone have been separated from the jaw, and if any are found they should be carefully removed, but if the gums interfere to prevent, they must be cut deeper so that the splinters may be successfully taken out.

The marks by which we recognize whether the jaw bone has been hurt or broken are when there is unusual bleeding from the gap from which the tooth has been taken, and such a swelling of the parts covering the jaw as to prevent one's opening the mouth, and the socket begins to fester and suppurate.

If no other damage appears put cold vinegar in the mouth in which gall-nuts and pomegranate flowers have previously been boiled.

Some being averse to extraction, used to burn the offending tooth as follows: They take a fine iron adapted to the purpose and make it red hot, then take a small iron tube prepared to receive the hot piece of iron without warming it up, then inserting the hot iron, which protrudes a little at the lower end, the bad tooth is burnt. But if the tooth is hollow they insert the red hot iron into that hollow. Such burning is very good and not dangerous, for it effects relief from pain, and causes the tooth by and by to crumble away without causing any pain. Some again instead of iron take the kernel of a nut, or a little olibanum, making this red hot and putting in the hollow or cavity of the tooth. Or take the fat of a lean frog and rub it on the tooth which will break it and cause it to fall out without any pains.

### THE LAST CHAPTER.

### HOW TO PRESERVE GOOD TEETH.

He who wishes to retain good teeth for a long time must refrain from things as above referred to in another chapter.

Every morning as soon as you have left your bed take a piece of rough linen, pass it over your teeth inwardly and outwardly, clean and rub them therewith once or twice. Such rubbing invigorates the teeth and gums, purifies and prevents putridity. Then take salt and rub likewise, and you shall keep your teeth white, fresh, firm and healthy.

Or take salt and honey mixed together in a pot, burn it to powder and therewith rub the teeth.

Or take myrrh and alum nicely powdered and rub the teeth therewith.

Or take burnt alum mixed with vinegar, wash your mouth therewith.

Or take myrrh boiled in wine, wash your mouth therewith. This strengthens the teeth and preserves the gums and prevents them from becoming stinky, and removes humidity.

In fine, after eating rinse your mouth with some wine or beer so as to remove every thing liable to attach to the teeth, and what would make them putrid and stinking and spoil them.

---

Haughtiness, Magnificence and Pride will be removed by bad luck, [adversity].

MAYENCE, BY PETER JORDAN, OF THE LEATHER BREECHES, IN THE YEAR 1532.

